# A Pharus Method for Laparoscopically Locating a Uterine Niche in Cesarean Scar Disorder

**DOI:** 10.1111/ases.70197

**Published:** 2025-12-02

**Authors:** Akiko Yoshida‐Ueno, Takayuki Sato, Kazutoshi Hayashi, Shinya Wakatsuki, Takaomi Namba, Yuji Tanaka, Shunichiro Tsuji

**Affiliations:** ^1^ Department of Obstetrics and Gynecology Kochi Health Sciences Center Kochi Japan; ^2^ Graduate School of Integrated Arts and Sciences, Medicine Program (Doctoral Course), Kochi Medical School Nankoku Japan; ^3^ Department of Cardiovascular Control Kochi Medical School Nankoku Japan; ^4^ Department of Obstetrics and Gynecology Shiga University of Medical Science Japan

**Keywords:** cesarean scar disorder, laparoscopic niche repair, Pharus method

## Abstract

**Introduction:**

Cesarean scar disorder (CSDi) is a pathological entity involving a myometrial defect at the site of a previous cesarean section, commonly associated with postmenstrual spotting, dysmenorrhea, and secondary infertility. While laparoscopic repair is an established treatment, intraoperative localization of the niche remains challenging.

**Materials and Surgical Technique:**

We report a novel application of the Pharus method, which utilizes the full‐color IR white‐light overlay mode of a commercial imaging platform (ELITE III, Olympus, Tokyo, Japan) to enable real‐time visualization of hysteroscopic transillumination as green lights under bright‐field conditions. A 32‐year‐old woman diagnosed with CSDi underwent combined hysteroscopic and laparoscopic niche repair. The margins of the niche were precisely delineated, sharply excised, and the uterine wall was reconstructed in two layers using barbed sutures.

**Discussion:**

This is the first reported use of the Pharus method in the laparoscopic repair of CSDi. The technique enhances intraoperative anatomical guidance without the need for reduced lighting. This method may offer a valuable advancement in laparoscopic repair of CSDi.

## 1 Introduction

Cesarean scar disorder (CSDi) is defined as an indentation of ≥ 2 mm in the myometrium at the site of a previous cesarean section scar, as identified by transvaginal ultrasonography, and accompanied by symptoms such as postmenstrual spotting, dysmenorrhea, chronic pelvic pain, or secondary infertility [1, 2, 3]. Due to its adverse impact on both quality of life and reproductive outcomes, minimally invasive laparoscopic repair has become a preferred therapeutic option [4].

In this report, we describe a novel surgical approach using the Pharus method [5], which utilizes the full‐color IR white‐light overlay mode of a commercially available endoscopic imaging platform (ELITE III, Olympus, Tokyo, Japan) to facilitate intraoperative identification and resection of the cesarean scar niche. This imaging modality converts visible hysteroscopic light into green light in real time without requiring a reduction in ambient lighting. As a result, thinned myometrial tissue can be accurately visualized and marked under standard bright‐field conditions, thereby enhancing procedural precision and safety.

The Pharus method was originally developed in our department for robot‐assisted sacrocolpopexy to improve anatomical orientation using near‐infrared (NIR) fluorescence imaging. In this technique, a transillumination light emitted from a rigid hysteroscope and passing through tissue is visualized as green light via NIR fluorescence imaging, providing real‐time visual guidance for the surgeon. To the best of our knowledge, this is the first report to apply the Pharus method to laparoscopic repair of CSDi.

## 2 Material and Surgical Technique

**FIGURE 1:**
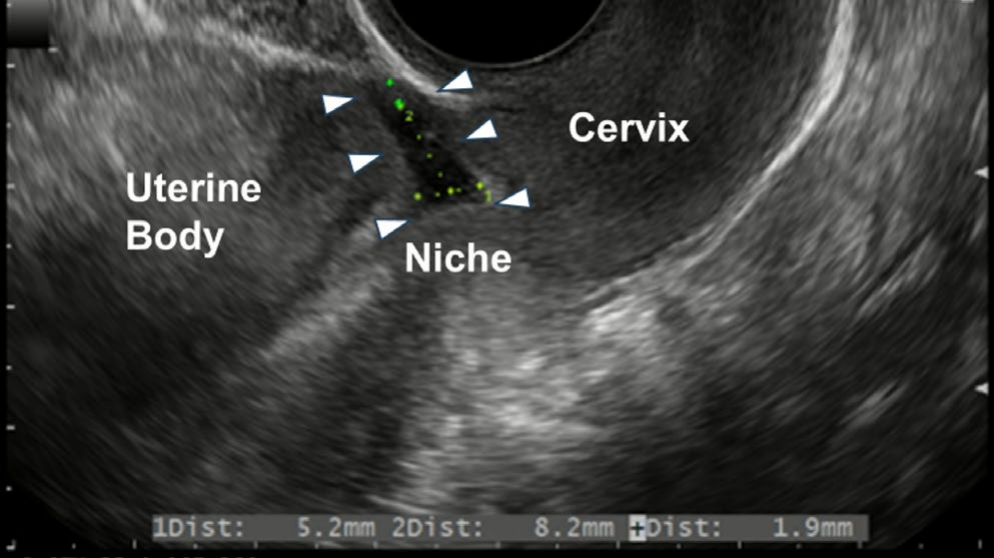
Imaging findings of a cesarean scar niche on transvaginal ultrasonography. Transvaginal ultrasonography identified a cesarean scar niche interposed between the uterine cervix and the uterine body, with dimensions of 5.2 mm in length and 8.2 mm in depth. The residual myometrial thickness (RMT) at the thinnest point measured 1.9 mm. These imaging findings were indicative of a cesarean scar disorder.

A 32‐year‐old woman with a history of three cesarean sections presented with dysmenorrhea, persistent postmenstrual brownish discharge, and chronic pelvic pain lasting for 4 years. She expressed a desire for future conception and requested surgical correction. Transvaginal ultrasonography revealed a cesarean scar niche measuring 5.2 mm in length and 8.2 mm in depth, with a residual myometrial thickness of 1.9 mm (Figure 1), consistent with a diagnosis of CSDi. Hormonal therapy, including a gonadotropin‐releasing hormone antagonist, had been discontinued 3 months prior to surgery.

A combined approach utilizing both hysteroscopic and laparoscopic techniques was employed. Hysteroscopy was performed using a 30°, 4‐mm rigid hysteroscope (OES ELITE WA2T430A; Olympus) mounted on a resectoscope (TCRis; Olympus). Laparoscopy was conducted using the 4K ELITE III system with 0° and 30°, 10‐mm laparoscopes.

Intraoperatively, moderate adhesions between the bladder and uterine serosa were observed and dissected without complication. The adnexae appeared grossly normal. During the hysteroscopic phase, the myometrium on the cervical side of the cesarean scar was incised to expose the niche. Multiple tortuous abnormal vessels were noted on the surface of the defect. The cervical and corporal margins were marked, and adjacent lateral and posterior myometrial tissue was coagulated using a ball electrode.

**FIGURE 2:**
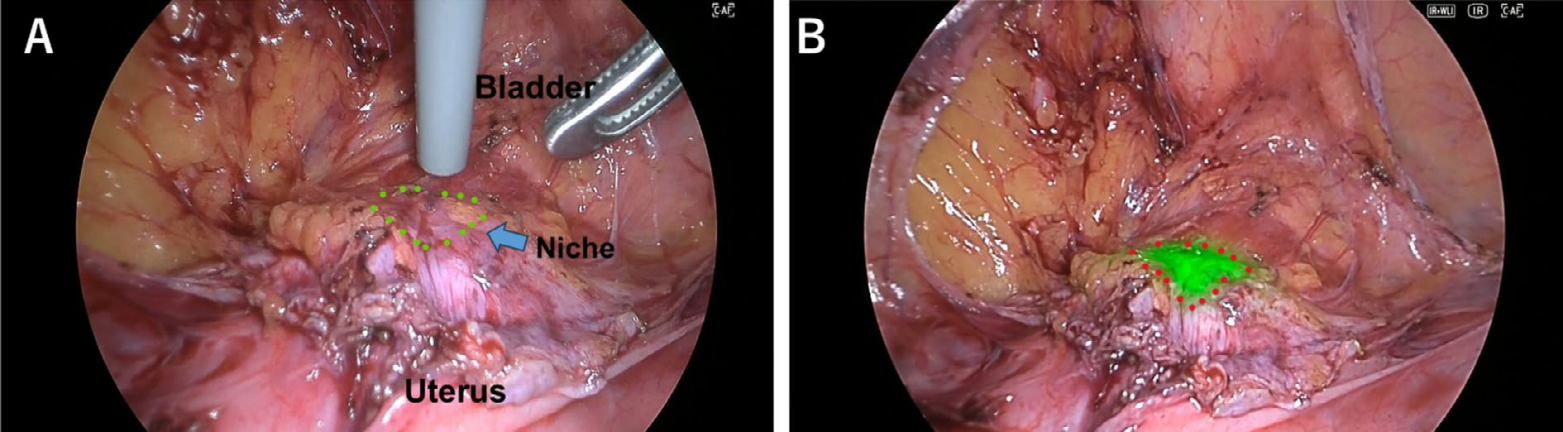
Laparoscopic identification of a cesarean scar niche using conventional white light and near‐infrared imaging with full‐color IR white‐light overlay mode (the Pharus method). (A) Normal white‐light laparoscopic view following completion of bladder dissection. The cesarean scar niche is indicated by green dots. Although a hysteroscope with its light source activated has been inserted into the uterine cavity, identification of the niche is challenging under conventional laparoscopic visualization alone. (B) Full‐color IR white‐light overlay mode of the near‐infrared imaging system (ELITE III) enabled. The light emitted from the hysteroscope is converted to green, allowing clear visual identification of the niche, which is delineated by red dots.

The laparoscopic phase commenced with dissection of the bladder flap. While maintaining hysteroscopic illumination, the laparoscopic camera was switched to full‐color IR white‐light overlay mode, with the gain set to its maximum level. Under standard bright‐field conditions, the light intensity was set to *Automatic mode at level 0* (within an adjustable range of −8 to +8), and transillumination through the thinned myometrium was visualized as green light, enabling precise delineation of the excision margins (Video S1, Figure 2). The correspondence between these green‐light boundaries and the hysteroscopically marked cervical and corporal edges was repeatedly confirmed by gentle probing with an electrocautery hook. The scar tissue was then sharply excised.

Uterine closure was performed in two layers, starting from the left lateral edge, using a barbed suture (V‐Loc M2115 GS‐22, Medtronic, Minneapolis, MN, USA). Although preoperative MRI demonstrated that the uterus was anteflexed, it tilted posteriorly toward the Douglas pouch intraoperatively after bladder dissection, likely due to the release of vesicouterine adhesions. To optimize uterine anteversion and to minimize tension on the surgical wound, only the right round ligament was plicated using a non‐absorbable suture (Ethibond Excel 2‐0, MX563, Ethicon, Johnson & Johnson, Somerville, NJ, USA). Following copious irrigation, an anti‐adhesion barrier was applied. Total operative time was 3 h and 12 min, and estimated blood loss was 10 mL. The postoperative course was uneventful, and the patient was discharged on postoperative day 4.

Histopathological examination of the excised tissue revealed endometrial glands and stroma embedded within the myometrium. Immunohistochemical staining demonstrated partial positivity for estrogen receptor (ER) and progesterone receptor (PgR), and CD10 positivity in stromal cells supported the diagnosis of endometriosis.

**FIGURE 3:**
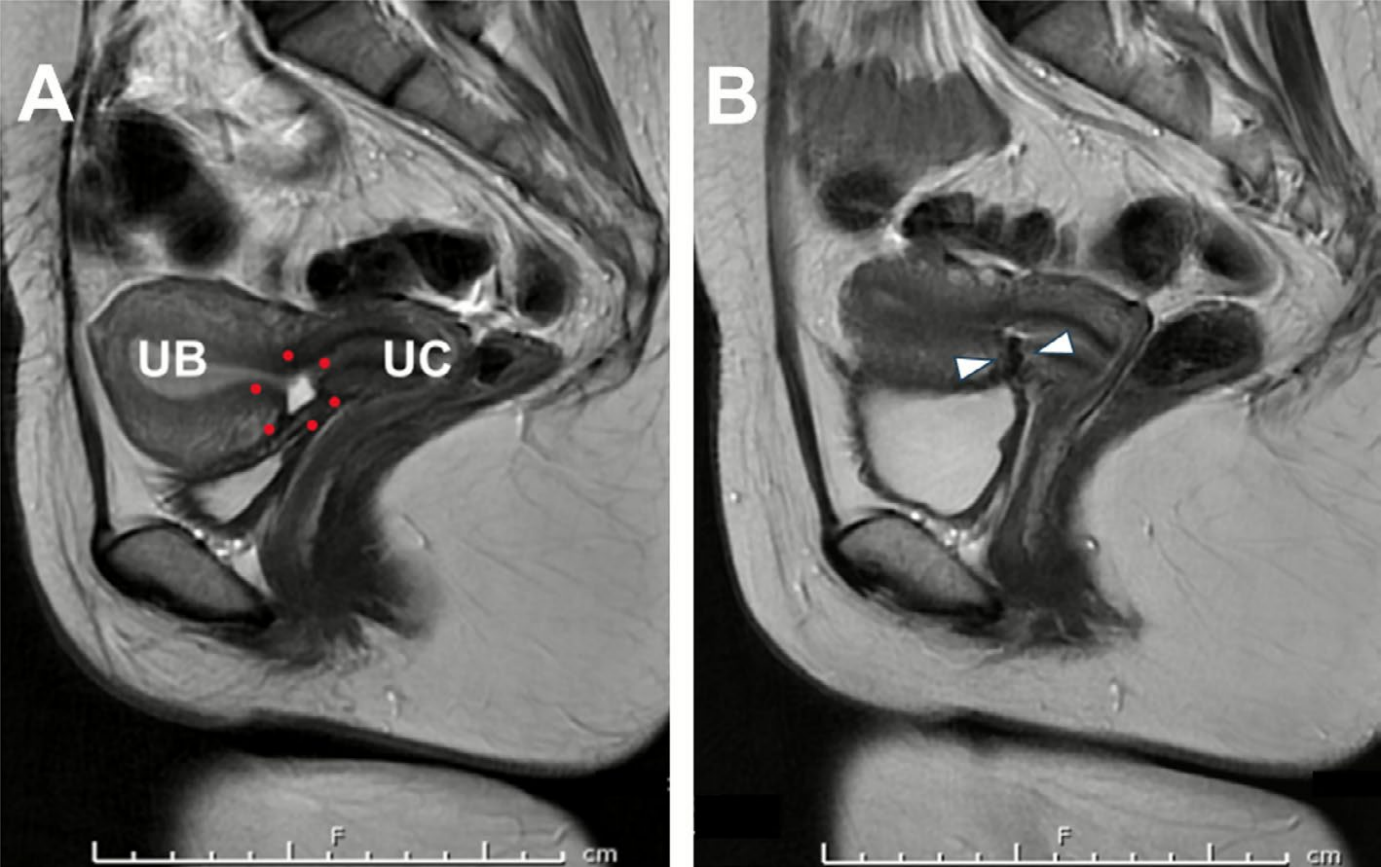
Preoperative and postoperative magnetic resonance imaging (MRI) findings. (A) Sagittal T2‐weighted image obtained from preoperative non‐contrast MRI. The anatomical landmarks are labeled as follows: uterine body (UB), uterine cervix (UC), and the cesarean scar niche, the latter being delineated by a red dotted outline. Consistent with the sonographic findings, the MRI delineated a pronounced niche formation accompanied by substantial thinning of the residual myometrium. (B) Sagittal T2‐weighted image obtained from postoperative non‐contrast MRI. The region corresponding to the cesarean scar niche observed in Image A is indicated by a white arrow; however, the niche has completely resolved, and the integrity of the myometrium is restored.

At the 3‐month follow‐up, non‐contrast‐enhanced magnetic resonance imaging confirmed complete resolution of the niche with preserved myometrial perfusion (Figure 3). Postoperative transvaginal ultrasonography revealed an increase in thickness of the myometrium at the site of the niche repair, from 1.9 to 9 mm. The primary symptom, namely postmenstrual spotting, completely resolved following the surgery. The patient was cleared for conception and continues to be monitored on an outpatient basis.

## 3 Discussion

Various intraoperative techniques have been described to enhance visualization of cesarean scar defects. One widely cited approach is the “Halloween sign,” introduced by Nirgianakis et al. [6], in which hysteroscopic light is projected into a darkened laparoscopic field to outline the scar. While effective, this technique requires a reduction in ambient lighting, which may compromise operative efficiency and continuity. Similarly, NIR imaging using the Firefly mode on the da Vinci Xi (intuitive surgical, California, USA) has been employed to visualize areas of myometrial thinning as green zones [7]; however, this also necessitates a dimmed operative field.

**FIGURE 4:**
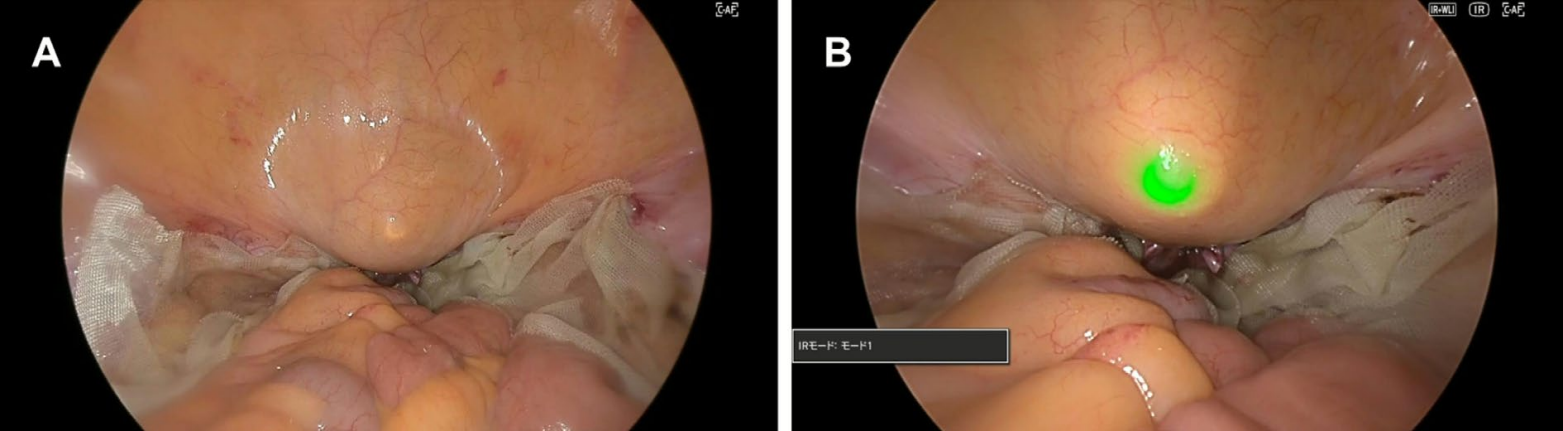
Enhanced visualization of rigid scope transillumination light using the full‐color IR white‐light overlay mode of the Olympus Elite III system. (A) Visualization of transmitted light from a 5‐mm rigid scope through the bladder wall under conventional white light during cystoscopic evaluation to exclude ureteral injury in laparoscopic hysterectomy. The transillumination light is faintly discernible through the bladder tissue. (B) Identical surgical field as in panel A, with the laparoscopic imaging system switched to full‐color IR white‐light overlay mode. The visible light transmitted through the bladder wall is converted to a green light by the near‐infrared imaging function of the Olympus Elite III system, thereby enhancing detectability.

Our technique overcomes these limitations by enabling transillumination‐based localization of the niche under standard lighting conditions. In our previous investigation of the Pharus method [5], we demonstrated that the Firefly imaging system is capable of converting not only NIR wavelengths but also visible light below 720 nm into green light, thereby enhancing the detectability of endoscopic light. To evaluate whether a similar optical conversion—specifically, the transformation of visible light into green via a NIR imaging modality—is feasible using the IR imaging function of the Olympus Elite III system, we conducted a validation study (Unpublished observation). During intraoperative cystoscopy performed at the conclusion of laparoscopic hysterectomy for the exclusion of ureteral injury, a rigid endoscope was utilized under the full‐color IR overlay mode (Figure 4). We confirmed that the endoscopic transillumination light through the bladder wall was captured as green light by the system, significantly enhancing its visibility. Based on these findings, we subsequently applied the Pharus method to the laparoscopic repair of CSDi.

In the present case, the boundary delineated by white light transillumination exhibited a strong correlation with the hysteroscopically defined margin. However, the question remains as to whether this correspondence can be universally applied. Therefore, at the current stage, it is essential to maximize light detection by setting the laparoscopic IR gain to its highest level and to verify the correspondence between the two boundaries by simultaneously observing the laparoscopic and hysteroscopic views. Further study is necessary to ascertain the efficacy of the Pharus method in laparoscopically locating the uterine niche.

This report presents the first successful application of the Pharus method for laparoscopic repair of CSDi. By utilizing the full‐color IR white‐light overlay mode to visualize hysteroscopic transillumination, this technique enables precise, efficient, and safe excision of scar tissue under full ambient lighting. We propose that this approach represents a meaningful advancement in the minimally invasive surgical management of CSDi.

## Ethics Statement

This study was approved by the Research Ethics Committee of our hospital. The patient's identity was protected.

## Consent

Written informed consent was obtained from the patient for publication of the accompanying images.

## Conflicts of Interest

The authors declare no conflicts of interest.

## Supporting information


**Video S1:** The procedure began with hysteroscopic part. Hysteroscopic findings revealed the interior of the cesarean scar niche. The upper surface exhibited a dome‐shaped protrusion toward the anterior uterine wall, and the niche surface was characterized by the presence of abnormal vessels. To enhance visualization of the cesarean scar niche, a portion of the myometrium on the cervical side was resected via hysteroscopy. The boundary between the cervical and corporal sides of the niche was marked, and the lateral and posterior myometrial margins within the planned resection area were cauterized using a ball electrode.
Subsequently, the procedure proceeded to the laparoscopic phase. After completion of bladder dissection, the *Pharus method* was applied, utilizing the full‐color IR white‐light overlay mode of the near‐infrared imaging system (ELITE III) to observe the transmitted light from the hysteroscope. This light was converted to green, thereby delineating the thinned area corresponding to the niche. To confirm anatomical accuracy, the boundary marked during hysteroscopy was gently probed using the hook of an electrosurgical device, while simultaneously verifying its correspondence with the green overlay under laparoscopic and hysteroscopic guidance.
The resection margins of the niche were then marked laparoscopically, and the niche was excised. Additional cauterization was performed using bipolar forceps on areas that were difficult to treat hysteroscopically. Two‐layer suturing was performed using barbed sutures, starting from the left resection margin. After plication of the right round ligament, symmetric anteflexion of the uterus was confirmed. The pelvic cavity was thoroughly irrigated, followed by application of an anti‐adhesion spray. The procedure was concluded without complication.

## Data Availability

The data that support the findings of this study are available on request from the corresponding author. The data are not publicly available due to privacy or ethical restrictions.

## References

[1] S. Tsuji , Y. Nobuta , T. Hanada , et al., “Prevalence, Definition, and Etiology of Cesarean Scar Defect and Treatment of Cesarean Scar Disorder: A Narrative Review,” Reproductive Medicine and Biology 22 (2023): e12532. 37577060 10.1002/rmb2.12532 PMC10412910

[2] O. Donnez , “Cesarean Scar Disorder: Management and Repair,” Best Practice & Research. Clinical Obstetrics & Gynaecology 90 (2023): 102398. 37598564 10.1016/j.bpobgyn.2023.102398

[3] S. J. M. Klein Meuleman , A. Murji , T. van den Bosch , et al., “Definition and Criteria for Diagnosing Cesarean Scar Disorder,” JAMA Network Open 6 (2023): e235321. 36988956 10.1001/jamanetworkopen.2023.5321 PMC10061236

[4] N. Barbany‐Freixa , Y. Hurni , M. Pellisé‐Tintoré , B. Graupera , S. Cabrera , and P. N. Barri‐Soldevila , “Long‐Term Anatomical and Functional Outcomes Following Laparoscopic Repair of Severe Cesarean Scar Defect,” Journal of Minimally Invasive Gynecology 32 (2025): 800–806. 40543760 10.1016/j.jmig.2025.06.011

[5] A. Yoshida‐Ueno , T. Sato , M. Kobayashi , S. Wakatsuki , T. Namba , and K. Hayashi , “Close Contact Transillumination Light Guides Surgeon to Vaginal Point Aa: Pharus Method for Robot‐Assisted Sacrocolpopexy,” Asian Journal of Endoscopic Surgery 18 (2025): e13412. 39578885 10.1111/ases.13412 PMC11584554

[6] K. Nirgianakis , R. Oehler , and M. Mueller , “The Rendez‐Vous Technique for Treatment of Caesarean Scar Defects: A Novel Combined Endoscopic Approach,” Surgical Endoscopy 30 (2016): 770–771. 26104791 10.1007/s00464-015-4226-6

[7] Z. Walker and A. Gargiulo , “Near‐Infrared and Hysteroscopy‐Guided Robotic Excision of Uterine Isthmocele With Laser Fiber: A Novel High‐Precision Technique,” Fertility and Sterility 120 (2023): 1081–1083. 37567494 10.1016/j.fertnstert.2023.08.006

